# Video-assisted thoracoscopic resection of a giant esophageal schwannoma: A case report

**DOI:** 10.1016/j.ijscr.2021.106202

**Published:** 2021-07-16

**Authors:** Usman Khan, Carmine Simone, Najib Safieddine, Sayf Gazala

**Affiliations:** a Division of General Surgery, Department of Surgery, University of Toronto, Toronto, Canada; b Division of Thoracic Surgery, Department of Surgery, University of Toronto, Toronto, Canada; c Division of Thoracic Surgery, Michael Garron Hospital, Toronto, Canada

**Keywords:** Esophagus, Schwannoma, VATS, Case report

## Abstract

**Introduction and importance:**

Intrathoracic schwannomas are rare and difficult to diagnose. However, they are the most common type of neurogenic tumor in the chest. Most patients are incidentally diagnosed or develop symptoms from mass effect, such as chest pain, dysphagia or dyspnea. Larger tumors have been resected using open approaches, while smaller ones are often excised with minimally invasive approaches.

**Case presentation:**

A 60-year-old woman with a prior Roux-en-Y gastric bypass and a history of dysphagia, decreased appetite, and weight loss was referred for evaluation. CT chest revealed an 8 cm soft tissue mass centered in the distal esophagus. Gastroscopy showed the tumor to be 8 cm as well, with 2 cm of normal esophagus prior to the gastric pouch. A right-sided video-assisted thoracoscopic (VATS) approach for enucleation was successfully completed with primary esophageal repair for an 8.0 × 5.5 × 6.5 cm schwannoma.

**Clinical discussion:**

Surgical resection for schwannomas is often indicated due to symptoms from mass effect (Moro et al., 2017). There are reports of VATS and robotic-assisted thoracic surgery approaches for small tumors. These techniques are appealing due to shorter length of stays and less post-operative pain. None have been described for lesions larger than 6 cm.

**Conclusion:**

Minimally invasive approaches such as VATS for large schwannomas are technically feasible and safe to perform without the need for a thoracotomy.

## Highlights

• Intrathoracic schwannomas are the most common type of neurogenic tumor in the chest.
• Schwannomas resected using video-assisted thoracoscopic surgery (VATS) are rare.
• Smaller esophageal schwannomas (<6 cm) have previously been approached using VATS.
• VATS can also be technically feasible and safe for larger lesions (8 cm).

## 1 Introduction

About 2% of esophageal tumors are benign [1]. Leiomyomas comprise of about 80% of these benign lesions, while schwannomas are very rare [2]. Establishing a pre-operative diagnosis is quite challenging and surgical excision is often indicated [3], [4]. There are less than 10 reported cases of esophageal schwannoma resected by video-assisted thoracic surgery (VATS) in literature [3], [5]. Amongst these reported cases, a mini-axillary thoracotomy has been often used for tumors larger than 50 mm [6], [7]. The largest tumor resected without a thoracotomy is by Onodera et al. who used a combined endoscopic and thoracoscopic approach for a 60 mm tumor [8]. Here, we discuss a novel VATS resection of an 80 mm esophageal schwannoma without a thoracotomy in a 60-year-old woman with a prior bariatric gastric bypass. This work has been prepared according to the SCARE guidelines 2020 [9].

## 2 Presentation of case

**Fig. 1:**
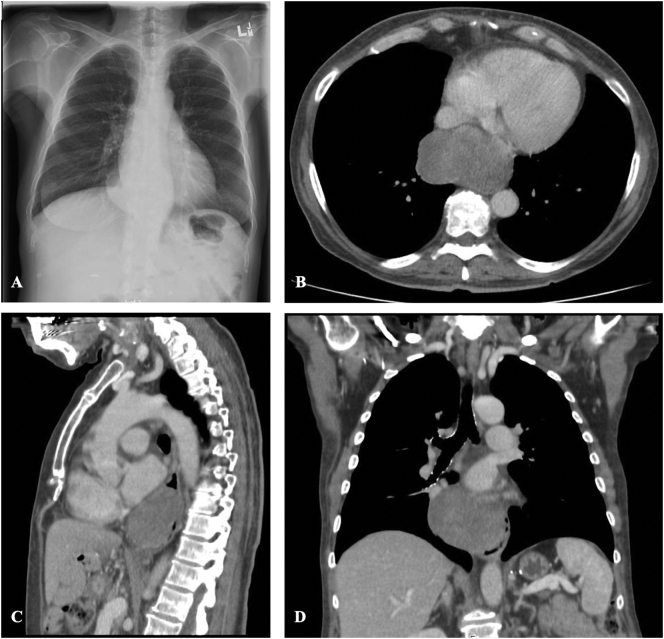
(A) Chest x-ray showing a large mediastinal mass. (B) Axial, (C) sagittal, and (D) coronal views of the chest computed tomography scan demonstrating the distal esophageal mass, 7.6 × 4.6 × 6.6 cm in size.

A 60-year-old woman with a history of a bariatric gastric bypass was referred for thoracic surgery assessment with symptoms of dysphagia, decreased appetite, and weight loss for several months. She was investigated with a chest x-ray (Fig. 1) and echocardiogram, which demonstrated a large mediastinal mass. Her CT chest revealed a soft tissue mass centered in the distal esophagus, measuring 7.6 × 4.6 × 6.6 cm (Fig. 1).

**Fig. 2:**
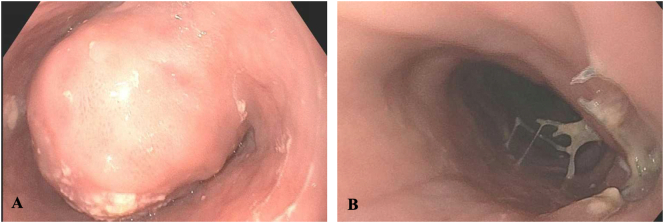
(A) Pre-operative endoscopy showing a large mass narrowing the lumen of the distal esophagus. (B) Post-resection endoscopy demonstrating a well-healed and patent esophagus.

On gastroscopy, the tumor was identified starting at 30 cm extending to 38 cm (Fig. 2). There was approximately 2 cm of normal esophagus before the GE junction which led to the gastric pouch. Initial endoscopic biopsies were non-diagnostic.

**Fig. 3:**
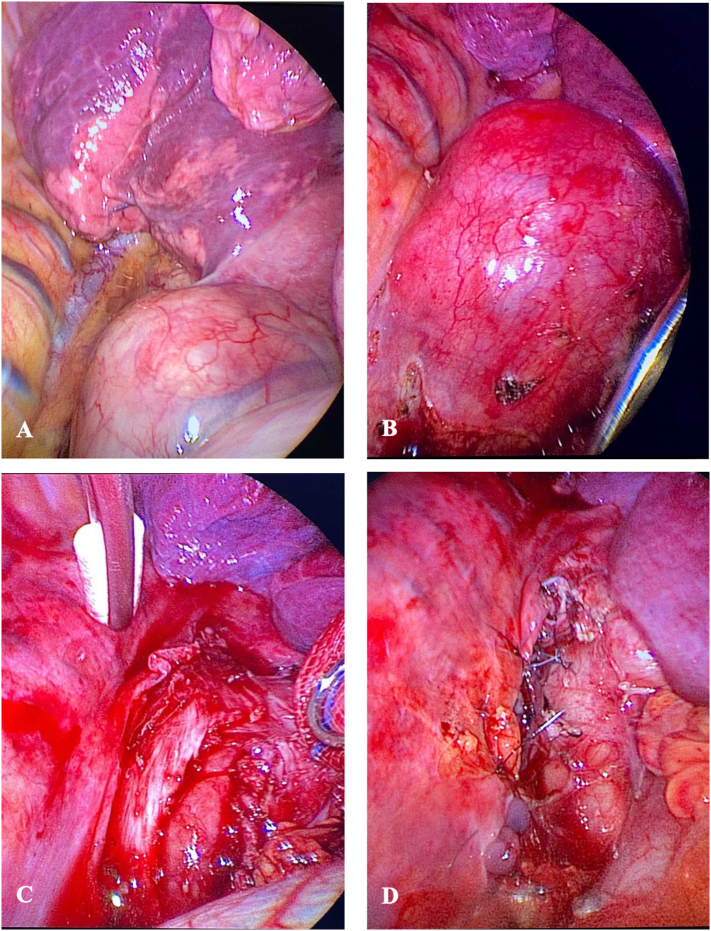
Thoracoscopic views. (A) Prior to dissection, the large tumor above the diaphragm and in close proximity to the inferior pulmonary ligament; (B) during dissection, given the size it was difficult to retract the tumor posteriorly because of the spine; (C) during dissection, the esophageal muscular layer divided and the submucosa intact except at the proximal end the tumor still attached to the esophagus; (D) after complete resection and pleural coverage of the esophagus resection area.

Surgical resection was planned by two experienced thoracic surgeons. The procedure started with right-sided VATS approach for enucleation of the tumor knowing that a thoracotomy may be needed, and possibly esophageal resection given the large size and location (Fig. 3). As the dissection was carried out the tumor was seen invading the submucosa and adherent to the mucosa on the left side, which required resection of the side wall of the esophagus to ensure complete resection. Once free, the tumor was removed through the utility incision. The mucosa and submucosa were sutured together primarily over a bougie to prevent future stricture. Then, a second layer closure was performed with a pedicled pleural patch over the esophageal muscular layer.

The post-operative course was complicated by a small and contained leak that required no intervention. Pathologic evaluation of the specimen revealed a schwannoma (8.0 × 5.5 × 6.5 cm). A gastroscopy was performed 8 weeks postoperatively (Fig. 2). This demonstrated a well healed distal esophagus and a patent GE junction. The gastric pouch appeared normal. At this time, the patient was symptom free, tolerating a regular diet, and beginning to regain her normal weight.

## 3 Discussion

Esophageal schwannomas are rare neurogenic tumors [10]. Surgical resection is often indicated due to symptoms from mass effect, pathological concern, or growth of the tumor [1]. Previous studies have described the open thoracotomy approach for resection of large schwannomas [2]. There are reports of VATS and robotic-assisted thoracic surgery (RATS) approaches for smaller tumors, often less than 2 cm in size [2], [11]. The latter techniques are attractive due to shorter length of stays and less post-operative pain. We describe a novel case of a VATS approach for a giant 8 cm distal esophageal schwannoma invading the submucosa.

This was a particularly challenging case given that the patient had a prior Roux-en-Y gastric bypass, limiting our surgical options. We offered a right-sided VATS with segmental resection. We did discuss that this option may not be feasible given the size of the mass and our ability to manipulate it. In that case, we would convert to a laparotomy and right-sided thoracotomy to complete an Ivor-Lewis esophagectomy. Given the size and location of the mass, our biggest challenge during the case was retracting the mass posteriorly. It became close to the spine, making our dissection around the inferior pulmonary ligament and the inferior pulmonary vein difficult. This required us to rotate the esophagus and complete the dissection from the contralateral side.

## 4 Conclusion

In this case report, we discuss a successful VATS resection approach for a large distal esophageal schwannoma invading the submucosa. We demonstrate that minimally invasive approaches for larger schwannomas are also feasible.

## Consent

Written informed consent was obtained from the patient for publication of this case report and accompanying images. A copy of the written consent is available for review by the Editor-in-Chief of this journal on request.

## Provenance and peer review

Not commissioned, externally peer-reviewed.

## Ethical approval

This study is exempt from ethical approval from our institution.

## Funding

This research did not receive any specific grant from funding agencies in the public, commercial, or not-for-profit sectors.

## Guarantor

Dr. Sayf Gazala is the guarantor of this study.

## Research registration number

Not applicable.

## CRediT authorship contribution statement

SG and CM performed the surgery. UK prepared the manuscript. SG, CM, NF reviewed and edited the manuscript for final submission.

## Declaration of competing interest

There are no conflicts of interest.
